# DNA methylation signatures in cord blood associated with maternal gestational weight gain: results from the ALSPAC cohort

**DOI:** 10.1186/1756-0500-7-278

**Published:** 2014-05-02

**Authors:** Eva Morales, Alexandra Groom, Debbie A Lawlor, Caroline L Relton

**Affiliations:** 1 Institute of Genetic Medicine, Newcastle University, Newcastle upon Tyne, NE1 3BZ, Tyne and Wear, UK; 2 Centre for Research in Environmental Epidemiology (CREAL), Barcelona, Catalonia, Spain; 3 Hospital del Mar Medical Research Institute (IMIM), Barcelona, Catalonia, Spain; 4 CIBER in Epidemiology and Public Health (CIBERESP), Barcelona, Spain; 5 MRC Integrative Epidemiology Unit at the University of Bristol, Bristol, UK; 6 School of Social and Community Medicine, University of Bristol, Bristol, UK

**Keywords:** DNA methylation, Epigenetics, Gestational weight gain

## Abstract

**Background:**

Epigenetic changes could mediate the association of maternal pre-pregnancy body mass index (BMI) and gestational weight gain (GWG) with adverse offspring outcomes. However, studies in humans are lacking. Here, we examined the association of maternal pre-pregnancy BMI and GWG in different periods of pregnancy with cytosine-guanine (CpG) dinucleotide site methylation differences in newborn cord blood DNA from 88 participants in the Avon Longitudinal Study of Parents and Children (ALSPAC) cohort using the Illumina GoldenGate Panel I. Pyrosequencing was used for validation of the top associated locus and for replication in 170 non-overlapping mother-offspring pairs from the ALSPAC cohort.

**Results:**

After correction for multiple testing greater GWG in early pregnancy (between 0 to 18 weeks of gestation) was associated with increased DNA methylation levels in four CpG sites at *MMP7*, *KCNK4*, *TRPM5* and *NFKB1* genes (difference in methylation >5% per 400 g/week greater GWG) (q values 0.023 -0.065). Pre-pregnancy BMI and GWG in mid- or late pregnancy were not associated with differential DNA methylation at any CpG site. Pyrosequencing showed that greater GWG in early pregnancy was associated with increased DNA methylation levels at the top associated CpG site at *MMP7*, although association did not reach statistical significance (p = 0.302). Greater GWG in mid- (p = 0.167) and late-pregnancy (p = 0.037) were also associated with increased DNA methylation levels at the *MMP7* CpG site. In addition, newborns of mothers who exceeded the IoM-recommended GWG had higher DNA methylation levels at the *MMP7* CpG site than those of mothers with IoM-recommended GWG (p = 0.080). We failed to replicate findings.

**Conclusions:**

Greater GWG in early pregnancy was associated with increased methylation at CpG sites at *MMP7*, *KCNK4*, *TRPM5* and *NFKB1* genes in offspring cord blood DNA. The specific association of GWG in early pregnancy with the top associated CpG site at *MMP7* was not validated using Pyrosequencing and it did not replicate. However, given the potential functional relevancy of the four identified loci, we advocate further exploration of them in larger studies.

## Background

Greater maternal pre- or early- pregnancy body mass index (BMI) and gestational weight gain (GWG) have been shown to be associated with health outcomes in offspring, including increased birth weight [1,2], impaired cognitive development and behavioral problems [3,4], increased adiposity and adverse cardiometabolic traits [5-13]. The biological mechanisms underpinning these associations remain elusive. Potential mechanisms may include genetic factors, shared postnatal environment, as well as intrauterine metabolic programming, possibly through epigenetic mechanisms [13,14]. With respect to potential intrauterine mechanisms the main exposure is greater maternal adiposity during pregnancy, and thus one might expect a specific association of GWG in early pregnancy, when the contribution of maternal fat deposition is greater than that of fetal weight or other GWG contributions in later periods, and little or no association of GWG in mid or later pregnancy with offspring outcomes [8,14]. In relation to offspring adiposity and cardiometabolic outcomes results from the cohort used in this study support this expectation [8].

To our knowledge only one study in humans has examined the association of maternal pregnancy adiposity with offspring DNA methylation patterns. In that study, cord blood white cell DNA methylation was compared between 25 offspring born before their mothers had undergone bypass surgery for severe obesity with 25 matched siblings born after marked weight loss by their mothers following bypass surgery, using the Illumina HumanMethylation450 BeadChip array [15,16]. Altered methylation and gene expression profiles of genes with known glucose-metabolic and inflammatory related functions were identified between those offspring born after their mothers surgery compared to those born before [15,16]. Recently a study conducted in African-American population has reported maternal pre-pregnancy BMI to be associated with offspring cord blood DNA methylation levels of the CpG sites in genes involved in a broad array of chronic diseases including cancer and cardiovascular diseases [17]. However, whether similar associations would be found between maternal GWG in humans is currently unknown. Nonetheless evidence from animal models strongly supports an effect of greater maternal pregnancy adiposity on offspring epigenome. For example, gene expression and DNA methylation patterns at loci involved in adipocyte commitment have been shown to be perturbed in rodent models of maternal obesity and overfeeding during pregnancy [18,19].

We hypothesize that maternal pre-pregnancy BMI and GWG, specifically in early pregnancy, could influence risk of adverse health outcomes in offspring by inducing epigenetic changes. Thus, we aimed to examine whether maternal pre-pregnancy BMI and GWG in different periods of pregnancy were associated with epigenetic marks in newborns, in terms of gene-specific cord blood DNA methylation, and to compare the early pregnancy GWG associations with mid- and late-pregnancy GWG to test whether there was any evidence of specific early pregnancy associations. Our analyses were undertaking in a discovery sample and then in a replication sample, both taken from a large, longitudinal UK cohort of mothers and their offspring.

## Method

### Discovery and validation study

#### Study population

Data were available from participants in the Avon Longitudinal Study of Parents and Children (ALSPAC), a prospective, population-based mother-child cohort study that recruited over 14 000 pregnant women resident in Avon, UK, between 1991 and 1992 (http://www.alspac.bristol.ac.uk) [20,21]. A subset of 96 children from the ALSPAC cohort was selected for analysis of DNA methylation at Cytosine-Guanine (CpG) dinucleotide sites in DNA extracted from cord blood samples. Samples used in this study had been analyzed as part of a study of maternal exposure to paracetamol, as described in previous literature [22], and were broadly representative of the ASPAC cohort. Of the 96 mother-offspring pairs with methylation data available, complete data on maternal pre-pregnancy BMI, GWG and potential confounders were available for 88 pairs.

#### Maternal pre-pregnancy BMI and GWG

Six trained research midwives abstracted data from obstetric medical records for the entire ALSPAC cohort. Obstetric data abstractions included every measurement of weight entered into the medical records and the corresponding gestational age and date. All pregnancy weight measurements (median number of repeat measurements per woman, 10; range, 1, 17) were used to develop a linear spline multilevel model (with 2 levels: woman and measurement occasion) relating gestational age to weight. Using the entire cohort of women with term pregnancies (≥37 weeks of gestation) fractional polynomial curves were fitted to the data to obtain the average shape of the trajectories of GWG with gestational age (for full statistical methodological details see [8]). These were used to determine the approximate positions of knots (indicating changes in slope) in linear spline random effects models with GWG as outcome and gestational age in weeks as the exposure. The knots produced from the modelling resulted in 4 variables: Pre-pregnancy weight (kg), change in weight between 0 and 18 weeks (kg/week), change in weight between 19 and 28 weeks (kg/week), and change in weight between 29 weeks and birth (kg/week). Pre-pregnancy body mass index (BMI) (kg/m^2^) was based on the mother’s self-reported weight before pregnancy and maternal report of height at their first questionnaire assessment (~12-18 weeks gestation) (results were identical for predicted pre-pregnancy weight with the use of multilevel models) [8]. To allocate women to Institute of Medicine (IoM) GWG categories (less than recommended, recommended, or more than recommended), we used weight measurements from the obstetric notes and subtracted the first from the last weight measurement in pregnancy to derive absolute weight gain [23].

#### Cord blood DNA methylation analysis using Illumina Golden Gate

DNA extracted from cord blood was used for DNA methylation analysis. Site-specific CpG methylation was analysed using bisulphite treated DNA (EZ-96 DNA Methylation Gold Kit, Zymo Research) using the the GoldenGate Cancer Panel I Array (Illumina Inc, USA) and the GoldenGate Assay Kit with UDG on the Sentrix Universal-96 Array matrix v7A. This panel spans 1,505 CpG sites selected from 807 genes. Arrays were imaged using a BeadArray scanner and image processing and intensity data extracted using Illumina BeadStudio v3.2, methylation module v3.2.5 custom software. Methylation levels (beta values, β) at a given CpG site were estimated as the ratio of signal intensity of the methylated alleles to the sum of methylated and unmethylated intensity signals of the alleles: β = M/(U + M), where M was the fluorescence level of the methylation probe and U was the fluorescence level of the unmethylated probe. The beta values vary from 0 (no methylation) to 1 (100% methylation). Samples were run across four different PCR plates.

#### Other variables

Based on previous knowledge, the following were considered a priori potential confounding factors because of their possible associations with either maternal pre-pregnancy BMI or GWG and DNA methylation levels: child’s sex and ethnic background, mode of delivery, maternal age at child’s birth, parity, maternal smoking in pregnancy and occupation. Child’s sex, maternal age, parity (0 or 1+) and mode of delivery (cesarean or vaginal delivery), and child’s ethnic background (white or non-white) were obtained from the obstetric records. Maternal smoking and occupational (of both the mother and her partner) were reported by the mother in a study questionnaire administered at mean 18 weeks gestation; with data on smoking also reported in subsequent questionnaires administered during pregnancy. Maternal smoking in pregnancy was categorized as no versus yes. Highest parental occupation was used to allocate the children to family social class groups according to the 1991 British Office of Population and Census Statistics classification [24] (with the higher class of either parent where these differed being used); this was collapsed into a binary variable of manual versus non-manual in this study.

#### Validation study

A PyroMark MD Pyrosequencing System (Qiagen) was used for the validation of the top associated locus (MMP7_E59). This quantifies DNA methylation in a sequencing-by-synthesis manner providing precise methylation levels of several CpG positions in close proximity [25]. The DNA segment harboring the region of *MMP7* amplified for this purpose and analyzed consisted of the sequence 5′- AGGCTGAGAAGCTATATAAATTTCTGCAGTCACTAGCAGAAAACACCAAATCAACCATAGGTCCAAGAACAATTGTCTCTGGA*CG*GCAGCTATG*CG*ACTCAC ***CG*** TGCTGTGTGCTGTGTGCCTGCTGCCTGGCAGCCTGGCCCTGCCGCTGCCTCAGGAGGCGGGAGGCATGAGTGAGCTACAGTGGGAAC-3′. As indicated in italics, we analyzed three CpG sites overall. The one referring to CpG probe site MMP7_E59 is highlighted in bold. The forward PCR and reverse PCR, and sequencing primers are AGGTTGAGAAGTTATATAAA, ATTCCCACTATAACTCACT, and AATTAATTATAGGTTTAAGA, respectively. Briefly, bisulfite conversion of genomic DNA was performed using EZ DNA Methylation Gold™ kit (Zymo Research) following the manufacturer’s protocol. Quantitative bisulphite Pyrosequencing (Qiagen, UK) with Pyro Q-CpG™ Software (version 1.0.6.) was subsequently used to determine the percentage methylation at individual CpG sites. 1ug of DNA was bisulfite modified. Bisulfite treated DNA was added to the first PCR reaction with 12.5 μl Hot Star Taq mastermix (Qiagen) and optimised primer concentrations and annealing temperature. PCR cycling conditions were: 95 degrees C for 15 min, 50 cycles of 95 for 15 secs, 45 degrees C for 30secs, 72 for 15 secs, 72 for 5 min, and 4 for hold/storage. Assays were assessed for amplification bias and reliability as described previously [26,27]. Zero and 100% in vitro methylated controls were run routinely alongside samples as internal controls, as well as, negative controls consisting of DNA free wells. Zero and 100% in vitro methylated internal controls showed good correlation (R^2^ = 0.99). Two independent replicates per sample were processed on separate runs, giving good correlation (R^2^ between 0.658 and 0.847).

### Replication study

The same Pyrosequencing assay used during the validation study of the top associated locus (MMP7_E59) was used to analyze 192 non-overlapping mother-offspring pairs randomly selected from the 2,183 ALSPAC cohort participants with cord blood DNA available and who were not included in the discovery study. Of these 192 pairs 170 (88%) could be successfully characterized.

### Statistical analysis

In the discovery study mixed linear regression models were fitted to estimate the association of pre-pregnancy BMI and GWG in different periods of pregnancy with DNA methylation in offspring cord blood, regressing the methylation status at each CpG site quantified by the Illumina beta value (dependent variable) against maternal pre-pregnancy BMI or GWG (independent variables). Maternal pre-pregnancy BMI and GWG were scaled to be clinically meaningful, examining the variation in CpG site DNA methylation per 1 SD change of maternal pre-pregnancy BMI and per 400 g gain per week of gestation for GWG [8]. Final models were adjusted for child’s sex and maternal age at child’s birth included as fixed coefficients and a PCR plate effect as a random batch coefficient. Allowance for other possible confounding variables such as child’s ethnic background, mode of delivery, parity, maternal smoking in pregnancy and occupation did not materially alter the estimates. Results were summarized as means and standard errors. Lists of CpG sites with differential methylation in beta values (difference >0.05) at a p value < 0.01– a pragmatic threshold for selecting CpG sites for further study – were generated for maternal pre-pregnancy BMI, and GWG in early (from 0 to 18 weeks of pregnancy), mid (from 19 to 28 weeks of pregnancy), and late pregnancy (from 29 weeks of pregnancy onwards). False discovery rate correction for multiple testing was performed and q-values were computed by the ‘qvalue’ package in the R statistical package version 2.9.1 (R Foundation for Statistical Computing, Vienna, Austria). A false discovery rate q-value of <0.1 was considered statistically significant.

In the validation and replication analyses based on Pyrosequencing, CpG site methylation levels were also analyzed by mixed linear regression models adjusted for child’s sex and maternal age at child’s birth as fixed coefficient and PCR plate as a random batch effect as outlined above.

### Ethical approval

Ethical approval for all aspects of data collection was obtained from the ALSPAC Law and Ethics Committee and the Local Research Ethics Committee in accordance with the guidelines of The Declaration of Helsinki. Written informed consent was obtained for all participants in the study.

## Results

### Discovery study: illumina golden gate

The discovery study population included 88 mother-offspring pairs. Participants were unrelated newborns born at term and 50% male. Mean (sd) maternal age at birth was 30.2 (3.5) years. In comparison with eligible participants for the present study, mothers included in the discovery study were older, and were less likely overweight or obese before pregnancy, but did not differ in GWG (Table 1).

**Table 1:** Characteristics of the study populations from the ALSPAC cohort

	**Discovery & Validation sample (n = 88)**^**†**^	**Replication sample (n = 170)**	**Rest of eligible (n = 1991)**^**§**^
**Child’s sex, male (%)**	51.1	50.6	49.5
**Age of mother at birth, mean (sd)**	30.6 (3.3)	28.7 (4.4)	27.4 (5.1)
**Maternal pre-pregnancy BMI (kg/m**^**2**^**)**			
Mean (sd)	22.2 (3.05)	23.8 (4.1)	23.1 (3.9)
Normal, 18.5-24.9, (%)	85.9	68.6	73.6
Underweight, < 18.5, (%)	1.2	2.4	4.8
Overweight, 25.0-29.9, (%)	10.6	20.7	15.5
Obese, ≥ 30, (%)	2.4	8.3	6.1
**GWG (kg/wk)**			
**0 to 18 weeks**			
Mean (sd)	0.32 (0.17)	0.31 (0.18)	0.30 (0.18)
Median	0.33	0.32	0.31
Min, Max	-0.33, 0.83	-0.22, 0.79	-0.64, 1.31
**19-28 weeks**			
Mean (sd)	0.55 (0.17)	0.54 (0.16)	0.53 (0.18)
Median	0.55	0.53	0.53
Min, Max	0.22, 1.00	0.14, 0.96	-0.18, 1.45
**29 weeks onwards**			
Mean (sd)	0.49 (0.18)	0.47 (0.20)	0.46 (0.21)
Median	0.47	0.45	0.45
Min, Max	-0.02, 0.95	0.03, 1.04	-0.54, 1.22
**IoM categories, (%)**			
Recommended	36.6	37.3	36.1
Less than recommended	34.1	31.9	35.8
More than recommended	29.3	30.8	28.1

^**†**^4 subjects initially included in the discovery study (n = 88) failed in the validation study.

^**§**^Participants of the ALSPAC cohort with available cord blood DNA.

Maternal pre-pregnancy BMI was not found to be associated with differentially methylated DNA at any CpG site in offspring cord blood (Additional file 1: Table S1).

Early pregnancy GWG was associated with differential DNA methylation at 44 CpGs sites in offspring cord blood (p value < 0.01) (Table 2). Six out of these 44 CpG sites (located in 5 genes) had a difference in the Illumina beta value of >0.05 (*MMP7*_ E59 p value *=* 1.55E-05*; KCNK4_P171* p value = 4.57E-05*; TRPM5_E87* p value = 0.0003*; NFKB1_P496* p value = 0.0002*; TRPM5_P721* p value = 0.0017*;* and *ABCC2_E16* p value = 0.0018); of these, 4 CpG sites remained robust to correction for multiple comparison testing: *MMP7_E59* (q value = 0.023), *KNCK4_P171 (q value = 0.034), TRPM5_E87* (q value = 0.065), and *NFKB1_P496* (q value = 0.065) (Table 2).

**Table 2:** List of CpG sites with differential methylation at a p value < 0.01 in newborn cord blood DNA per 400 g of weight gain/week in early pregnancy (from 0 to 18 weeks), the ALSPAC cohort (n = 88)

**Chr**^**a**^	**Gene name**	**CpG site**	**Position**^**b**^	**Coef**^**c**^**. (se)**		**p value**	**q value**
11	**Matrix metallopeptidase 7**	**MMP7_E59**	101906629	**0.072**	**(0.016)**	**1.55E-05**	**0.023**
11	**Potassium channel, subfamily K, member 4**	**KCNK4_P171**	63815280	**0.060**	**(0.014)**	**4.57E-05**	**0.034**
19	Janus kinase 3	JAK3_P1075	17820875	0.034	(0.008)	7.13E-05	0.036
11	**Transient receptor potential cation channel, subfamily M, member 5**	**TRPM5_E87**	2400764	**0.072**	**(0.020)**	**0.0003**	**0.065**
12	Protein tyrosine phosphatase, non-receptor type 6	PTPN6_E171	6926172	0.042	(0.011)	0.0003	0.065
4	**Nuclear factor of kappa light polypeptide gene enhancer in B-cells 1**	**NFKB1_P496**	103641022	**0.058**	**(0.016)**	**0.0002**	**0.065**
1	Absent in melanoma 2	AIM2_E208	157313063	0.015	(0.004)	0.0003	0.065
17	Ecotropic viral integration site 2A	EVI2A_E420	26672423	0.039	(0.010)	0.0002	0.065
17	Septin 9	SEPT9_P58	72827686	0.034	(0.010)	0.0005	0.077
15	Interleukin 16	IL16_P93	79262162	0.037	(0.011)	0.0006	0.077
9	PALM2-AKAP2 protein	PALM2_AKAP2_P183	111582227	0.025	(0.007)	0.0005	0.077
20	DNA (cytosine-5-)-methyltransferase 3 beta	DNMT3B_P352	30813500	0.040	(0.012)	0.0005	0.077
13	5-hydroxytryptamine (serotonin) receptor 2A	HTR2A_E10	46368166	0.011	(0.003)	0.0008	0.096
6	RAB32, member RAS oncogene family	RAB32_P493	146906028	0.038	(0.011)	0.0009	0.098
22	Breakpoint cluster region (BCR)	BCR_P422	21852130	0.024	(0.007)	0.0010	0.103
9	Transmembrane protein with EGF-like and two follistatin-like domains 1	TMEFF1_P234	102275304	0.042	(0.013)	0.0016	0.146
11	Transient receptor potential cation channel, subfamily M, member 5	TRPM5_P721	2401572	0.053	(0.017)	0.0017	0.148
10	ATP-binding cassette, sub-family C (CFTR/MRP), member 2	ABCC2_E16	101532577	0.078	(0.025)	0.0018	0.148
12	v-Ki-ras2 Kirsten rat sarcoma viral oncogene homolog	KRAS_E82	25295039	0.042	(0.014)	0.0021	0.155
11	Tumor susceptibility gene 101	TSG101_P139	18505204	0.045	(0.015)	0.0022	0.155
11	Solute carrier family 22 (organic cation transporter), member 18	SLC22A18_P216	2877311	0.032	(0.011)	0.0021	0.155
20	v-src sarcoma (Schmidt-Ruppin A-2) viral oncogene homolog (avian)	SRC_P164	35406338	0.051	(0.017)	0.0027	0.177
6	Major histocompatibility complex, class II, DO beta	HLA_DOB_P1114	32893876	0.014	(0.004)	0.0027	0.177
X	Biglycan	BGN_P333	152413272	0.027	(0.009)	0.0030	0.181
2	Interleukin 1, beta	IL1B_P829	113311656	0.029	(0.009)	0.0030	0.181
11	Cathepsin D (lysosomal aspartyl peptidase)	CTSD_P726	1742524	0.033	(0.011)	0.0034	0.192
7	Carboxypeptidase A4	CPA4_P961	129719269	0.020	(0.007)	0.0035	0.192
22	Breakpoint cluster region (BCR)	BCR_P346	21852206	0.021	(0.007)	0.0036	0.192
16	PYD and CARD domain containing	PYCARD_E87	31121665	0.023	(0.008)	0.0038	0.192
17	Homeo box B2	HOXB2_P99	43977490	0.040	(0.014)	0.0038	0.192
17	v-crk sarcoma virus CT10 oncogene homolog (avian)	CRK_P721	1307015	0.043	(0.015)	0.0039	0.192
22	Seizure related 6 homolog (mouse)-like	SEZ6L_P299	24895181	0.031	(0.011)	0.0044	0.208
19	Kallikrein 11	KLK11_P103	56223205	0.022	(0.008)	0.0046	0.210
X	ELK1, member of ETS oncogene family	ELK1_E156	47394808	0.025	(0.009)	0.0058	0.255
2	CDC-like kinase 1	CLK1_P538	201438205	0.036	(0.013)	0.0064	0.263
6	Solute carrier family 22 (organic cation transporter), member 2	SLC22A2_P109	160600058	0.021	(0.008)	0.0064	0.263
19	Apolipoprotein C-II	APOC2_P377	50140706	0.015	(0.006)	0.0067	0.263
X	Dyskeratosis congenita 1, dyskerin	DKC1_P276	153644068	0.030	(0.011)	0.0066	0.263
20	v-src sarcoma (Schmidt-Ruppin A-2) viral oncogene homolog (avian)	SRC_P297	35406205	0.025	(0.009)	0.0080	0.300
10	N-acetyltransferase 2	NAT2_P11	18293024	0.012	(0.004)	0.0079	0.300
8	PTK2 protein tyrosine kinase 2	PTK2_P735	142081249	0.022	(0.008)	0.0085	0.312
1	CD34 antigen	CD34_P339	206151645	0.021	(0.008)	0.0092	0.330
4	Interleukin 8	IL8_P83	74825056	0.051	(0.019)	0.0098	0.334
11	Progesterone receptor	PGR_P790	100507255	0019	(0.007)	0.0096	0.334

^a^Chromosome. ^b^Chromosomal position based on NCBI human reference genome assembly Build 36.1.

^c^Regression coefficient using linear mixed models. All models adjusted for child’s sex and maternal age at child’s birth and the inclusion of a PCR plate random batch effect.

Suffixes denote the Illumina probe identity (P = within promoter, E = within first exon).

Probes shown in bold displayed >5% difference in methylation.

Greater GWG in mid pregnancy was associated with changes in methylation (difference in beta value >0.05) at one CpG site at *MAP3K1_E81* (p value = 0.003); although it did not remain statistically significant after correction for multiple comparison testing (q value = 0.917) (Additional file 1: Table S2). GWG in late pregnancy was not associated with differential DNA methylation at any CpG site (all differences in beta values <0.05) (Additional file 1: Table S3).

### Pyrosequencing validation study

The association of early pregnancy GWG with differential methylation at the top associated CpG site at *MMP7* was validated by reanalyzing CpG site *MMP7_E59* in the discovery study using Pyrosequencing. The methylation levels assayed using the GoldenGate platform did not correlate with Pyrosequenced values (R-squared = 0.005; p value = 0.508).

The two adjacent CpG sites covered in the Pyrosequenced amplicon were highly correlated with *MMP7_E59* and with each other (Pearson’s correlation coefficients all >0.87; all p < 0.0001). In agreement with the discovery findings, Pyrosequencing results showed that greater GWG in early pregnancy tended to be associated with higher methylation at the *MMP7_E59* CpG site, although the association did not reach statistical significance (p value = 0.302 (Table 3). Moreover, greater GWG in mid- and late pregnancy were associated with increased methylation at the *MMP7_E59* CpG site (p values = 0.167 and 0.037, respectively) (Table 3). In addition, newborns of mothers that gained greater than IoM recommended weight showed higher methylation levels at the *MMP7_E59* CpG site compared to those newborns of mothers with IoM recommended weight (p value = 0.080) (Table 3). Maternal GWG and IoM recommended weight categories showed similar associations with DNA methylation levels at the other 2 CpG sites evaluated at *MMP7*.

**Table 3:** **Validation study: associations of maternal pre-pregnancy BMI and gestational weight gain (GWG) with *MMP7 *** **CpG sites methylation levels in newborn cord blood DNA**

	**CpG1**		**CpG2**		**CpG3 (E59)**		**Mean of 3 CpGs**	
	**Coef**^**a **^**(se)**	**p value**	**Coef**^**a **^**(se)**	**p value**	**Coef**^**a **^**(se)**	**p value**	**Coef**^**a **^**(se)**	**p value**
**Pre-pregnancy BMI (n = 87)**	0.80 (0.70)	0.250	1.15 (0.78)	0.143	1.22 (0.82)	0.139	1.06 (0.75)	0.158
**GWG 0 to 18 wks (n = 84)**	0.80 (1.99)	0.686	1.52 (2.27)	0.505	2.44 (2.36)	0.302	1.59 (2.15)	0.461
**GWG 19 to 28 wks (n = 84)**	0.96 (1.73)	0.580	2.89 (1.95)	0.141	2.83 (2.04)	0.167	2.23 (1.86)	0.232
**GWG 29 wks. onwards (n = 84)**	2.38 (1.57)	0.133	3.25 (1.79)	0.071	3.90 (1.85)	0.037	3.17 (1.69)	0.062
**GWG IoM categories (n = 78)**								
Recommended	0 [Ref.]		0 [Ref.]		0 [Ref.]		0 [Ref.]	
Less than recommended	2.03 (1.74)	0.246	0.18 (2.00)	0.930	0.38 (2.09)	0.853	0.86 (1.90)	0.649
More than recommended	3.35 (1.79)	0.063	3.13 (2.06)	0.131	3.81 (2.16)	0.080	3.43 (1.96)	0.082

^a^Regression coefficient using linear mixed models. For pre-pregnancy BMI (kg/m2) estimated changes per 1 SD change and for GWG estimated changes per additional 400 g/week.

All models adjusted for child’s sex and maternal age and the inclusion of a PCR plate random batch effect.

### Replication study

The replication study population included 170 non-overlapping mother-offspring pairs from the ALSPAC cohort. Participants were unrelated newborns born at term and 51% male. Mean (sd) maternal age at birth was 28.7 (4.4) years. Compared with the discovery study sample those included in the replication study have younger mothers that were more likely overweight or obese before pregnancy, but did not differ in GWG (Table 1). Pyrosequencing results showed no association of maternal pre-pregnancy BMI or GWG in any period of pregnancy with differential methylation at *MMP7* CpG sites (all p values >0.1) (Table 4).

**Table 4:** **Replication study: associations of maternal pre-pregnancy BMI and gestational weight gain (GWG) with** ***MMP7*** **CpG sites methylation levels in newborn cord blood DNA (n = 170)**

	**CpG1**		**CpG2**		**CpG3 (E59)**		**Mean of 3 CpGs**	
	**Coef**^**a **^**(se)**	**p value**	**Coef**^**a **^**(se)**	**p value**	**Coef**^**a **^**(se)**	**p value**	**Coef**^**a **^**(se)**	**p value**
**Pre-pregnancy BMI**	-0.03 (0.57)	0.952	-0.44 (0.66)	0.501	-0.48 (0.69)	0.486	-0.31 (0.62)	0.613
**GWG 0 to 18 wks.**	-0.34 (1.28)	0.791	-0.62 (1.46)	0.671	-0.76 (1.55)	0.621	-0.58 (1.39)	0.674
**GWG 19 to 28 wks.**	-0.25 (1.45)	0.863	-1.04 (1.65)	0.528	-0.46 (1.75)	0.790	-0.60 (1.56)	0.702
**GWG 29 wks. onwards**	0.57 (1.16)	0.622	0.42 (1.33)	0.751	0.01 (1.41)	0.994	0.34 (1.26)	0.787
**GWG IoM categories**								
Recommended	0 [Ref.]		0 [Ref.]		0 [Ref.]		0 [Ref.]	
Less than recommended	-2.00 (1.37)	0.144	-1.54 (1.56)	0.323	-1.62 (1.65)	0.329	-1.71 (1.48)	0.249
More than recommended	-1.85 (1.39)	0.185	-2.07 (1.59)	0.195	-2.24 (1.68)	0.185	-2.04 (1.51)	0.177

^a^Regression coefficient using linear mixed models. For pre-pregnancy BMI (kg/m2) estimated changes per 1 SD change and for GWG estimated changes per additional 400 g/week.

All models adjusted for child’s sex and maternal age and the inclusion of a PCR plate random batch effect.

## Discussion

This study examined the association of maternal pre-pregnancy BMI and GWG in different periods of pregnancy with gene-specific DNA methylation changes in offspring cord blood. Screening analysis using the Illumina GoldenGate Panel I in a discovery sample of 88 mother-offspring pairs showed greater GWG in early pregnancy (from 0 to 18 weeks) to be associated with differential methylation at 4 CpG sites at *MMP7*, *KCNK4*, *TRPM5* and *NFKB1* genes after correcting for multiple statistical testing. Of these four loci we undertook validation and replication using Pyrosequencing of the top associated locus at *MMP7*. Results of the validation study did not support evidence for an association with early pregnancy GWG with differential methylation at this locus, but was suggestive of an association with GWG in later pregnancy and for exceeded IoM recommended GWG. However, we failed to replicate these findings for this one locus in 170 non-overlapping mother-offspring pairs of the ALSPAC cohort.

The specific association of early pregnancy GWG with differential methylation at four loci is notable, since early GWG is more likely to reflect maternal fat deposition than later GWG (which will be influenced more by fetal growth) [13], and so this specificity, together with the potential functional roles of these loci (see below), might support these findings as being important in developmental overnutrition. However, we were only able to undertake validation and replication studies for one, the most strongly associated loci (*MMP7*) and the specific early GWG association with differential methylation at that locus did not validate or replicate.

Our more significant finding in the discovery study was increased methylation of CpG sites at *MMP7* gene in relation to greater GWG. Matrix metalloproteinases (MMPs) comprise a large family of structurally related zinc-dependent proteinases that have been classified into subgroups on the basis of their structure, substrate specificity, and cellular localization and include collagenases, gelatinases, stromelysins, and membrane-type (MT-MMPs) [28]. MMPs participate in processes such as embryonic development, angiogenesis, wound repair, reproductive cycling, and metastasis [28]. Interestingly, MMPs are essential for proper extracellular matrix remodelling, a process that takes place during obesity-mediated adipose tissue formation. Specifically, mouse models of obesity have showed that the expression of *MMP7* is down regulated in obesity [29,30]. These findings are in accordance with our results since increased methylation of *MMP7* would imply lower gene expression, which could translate into a higher risk of adiposity in the offspring in response to an *in utero* obesogenic environment. However, these associations were not replicated in non-overlapping mother-offspring pairs; thus, present results should be interpreted cautiously and future studies are warranted to confirm the association of greater GWG with differential methylation of *MMP7*.

Validation and replication studies using Pyrosequencing were not undertaken for other CpG sites that were identified as being differentially methylated in relation to greater GWG and located in genes *KCNK4*, *TRPM5* and *NFKB1*. However, these three identified genes may be worthy of further exploration. *KCNK4* encodes for a potassium channel, subfamily K, member 4 known as Trek/TRAAK channels that has been described to be expressed in the central nervous system, modulate neuronal activity, brain metabolism and have a role in neuroprotection [31,32]. Knockout animal models have shown a role for Trek/TRAAK channels in behaviour, learning and memory [33,34]. Interestingly, greater maternal pre-pregnancy BMI and GWG have been shown to be associated with offspring impaired cognition and behavioural problems later in life, including in the ALSPAC study [3,4]. In addition, *TRPM5* encodes for a thermo-sensitive TRP (transient receptor potential) channel that is expressed in pancreatic β-cells and could predominantly contribute to pancreatic functions [35]. In humans, genetic variation in *TRPM5* has been reported to be likely associated with pre-diabetic phenotypes and contribute to the development of type 2 diabetes mellitus [36]. Furthermore, *TRPM5* knockout mice exhibit impaired glucose clearance resulting from reductions in insulin secretion [37,38]. This evidence from animal studies is in accordance with our results showing increased methylation of *TRPM5* CpG sites in relation to greater maternal GWG; this may in turn result in lower expression of *TRPM5* and, thus, perturbed insulin metabolism and increased risk of diabetes in the offspring as it has been previously suggested [39]. Finally, *NFKB1* encodes for nuclear factor of kappa light polypeptide gene enhancer in B-cells 1, which is responsible for activation of transcription of genes involved in immune responses, inflammation or cell proliferation [40]. Genetic variation in *NKKB1* gene has been reported to be associated with a range of immune-mediated diseases such as atopy, asthma and related phenotypes [41], which are also related to maternal obesity and GWG [42,43]. Therefore, DNA methylation changes at these three loci highlighted in the screening analysis appear to have clear functional relevance to pathways implicated in developmental programming of adverse phenotypes in offspring linked to maternal obesity and GWG and therefore deserve further investigation.

The strengths of this study are the prospective design and that potential confounding factors were taken into account in the analyses. Further, we evaluated the association of pre-pregnancy BMI and GWG in distinctive periods of pregnancy with offspring cord blood DNA methylation patterns. To our knowledge no previous human studies have examined these associations, yet these maternal exposures have been shown to be related to later offspring outcomes and it has been suggested these associations may be mediated by epigenetic mechanisms [8,13,14]. The ability to examine associations of different pregnancy periods of GWG is a particular strength as our expectation would be that GWG in early pregnancy would be specifically associated with outcomes and we were able to test that here.

However, the study has some limitations. Firstly, DNA methylation changes were assessed in cord blood, which limits to know how identified changes could translate to potential target tissues. However, DNA methylation patterns are largely conserved across tissues suggesting that for population based epidemiological studies methylation markers from easily accessible surrogate tissues could be used as a proxy for methylation in target tissues [44]. Because cell count information was not available we cannot rule out the potential impact of cell heterogeneity on present findings. Secondly, limited sample size of the discovery study population may have resulted in low statistical power to detect true differences in DNA methylation patterns and false positives could have occurred. In addition, lack of correlation of methylation levels in *MMP7* CpG sites measured with the GoldenGate array and Pyrosequencing limits the conclusions that can be drawn from the present validation and replication studies. Thirdly, smaller effect sizes in DNA methylation were found and biological significance in terms of changes in gene expression is unknown. However, it may be a general phenomenon in complex diseases and phenotypes, where methylation at any given CpG island or specific CpG sites in affected versus unaffected individuals may vary by less than 10%, indeed they have been reported in many other human epigenetic studies. Moreover, for some genes, evidence exists that a small change in the level of DNA methylation, especially in the lower range, can dramatically alter gene expression levels [45]. We opted not to consider differentially methylated site below a threshold of 5% due to the limitations inherent in interpreting biological significance, although this cut off may have limited our findings. Fourthly, the Illumina GoldenGate Panel I was used for screening of DNA regions differently methylated in DNA cord blood. The CpG sites included in this array are based on their functional relevance to tumor development and cancer processes, which in only some aspects can be related to fetal development (such as cell proliferation). However, this array was not designed for epigenome-wide analyses, and methylation changes at other loci in genes relevant to maternal GWG may have been overlooked. The use of the 450 k Beadchip that offers greatly improved genomic coverage over the GoldenGate and 27 k platforms is warranted in future studies. Finally, we were only able to take forward the top associated loci at *MMP7* for validation and replication, which limits the interpretation of results for the other three loci that were identified as being associated with GWG in early pregnancy. Given the likely functional relevance of these and the specificity of association with early GWG we feel that further study of these loci is warranted.

## Conclusions

We found that greater GWG, specifically in early pregnancy, was associated with increased methylation at 4 CpG sites at *MMP7*, *KCNK4*, *TRPM5* and *NFKB1* genes in offspring cord blood DNA. These four loci were all potentially functionally relevant but we were only able to take the top associated locus at *MMP7* forward for validation using Pyrosequencing and replication in non-overlapping mother-offspring pairs. The specific association with GWG in early pregnancy for that one site was not statistically significantly validated and it did not replicateple. Given this is the first study we are aware of to examine these associations and our findings might reflect limited statistical power, we advocate further exploration of identified loci in larger studies and the study of genome-wide DNA methylation data.

## Competing interests

The authors declare that they have no competing interests.

## Authors’ contributions

DAL and CLR: obtained funding for the study and initially designed it. EM and CLR: developed the study aims. EM and AG: conducted the experiments, under the supervision of CLR. EM: performed the statistical analyses and wrote the first draft of the manuscript. EM, DAL and CLR participated in the interpretation of data. All authors help to draft the manuscript, and read and approved the final manuscript.

## Supplementary Material

Additional file 1 Supplementary material for DNA methylation signatures in cord blood associated with maternal gestational weight gain: results from the ALSPAC cohort. Click here for file
