# Supplementary material for: DNA methylation signatures in cord blood associated with maternal gestational weight gain: results from the ALSPAC cohort
Source: BMC Res Notes. 2014 May 2;7:278. doi: 10.1186/1756-0500-7-278 (PMC4108052; doi:10.1186/1756-0500-7-278)
Supplement: Additional file 1 — Supplementary material for DNA methylation signatures in cord blood associated with maternal gestational weight gain: results from the ALSPAC cohort. [file 1756-0500-7-278-S1.doc]

**Supplementary material for**

**DNA methylation signatures in cord blood associated with maternal gestational weight gain: results from the ALSPAC cohort**

Eva Morales, Alexandra Groom, Debbie A. Lawlor, Caroline L. Relton

**Table S1.** List of CpG sites with differential methylation at a p value<0.01 per 1 SD change in maternal pre-pregnancy BMI (kg/m^2^), the ALSPAC cohort (n=84).

| **Gene name** | **CpG site** | **Mean (se) difference in Illumina beta value** | | **p value** | **q value** |
| --- | --- | --- | --- | --- | --- |
|  |  |  |  |  |  |
| Interleukin 8 precursor | IL8_E118 | -0.021 | (0.006) | 0.002 | 0.999 |
| Peptidyl arginine deiminase, type IV | PADI4_P1158 | -0.021 | (0.006) | 0.002 | 0.999 |
| CD82 antigen isoform 1 | CD82_P557 | -0.021 | (0.009) | 0.007 | 0.999 |
| LIM domain only 2 | LMO2_E148 | -0.018 | (0.002) | 0.009 | 0.999 |
|  |  |  |  |  |  |

Mean difference estimated using linear mixed models. All models adjusted for child’s sex and maternal age at child’s birth and the inclusion of a random batch effect.

Suffixes denote the Illumina probe identity (P = within promoter, E = within first exon).

**Table S2.** List of CpG sites with differential methylation at a p value<0.01 per 400g of weight gain/week in mid pregnancy (from 19 to 28 weeks), the ALSPAC cohort (n=88).

| **Gene name** | **CpG site** | **Mean (se) difference in Illumina beta value** | | **p value** | **q value** |
| --- | --- | --- | --- | --- | --- |
|  |  |  |  |  |  |
| Excision repair cross-complementing rodent repair deficiency, complementation group 3 | ERCC3_P1210 | -0.034 | (0.011) | 0.003 | 0.917 |
| Mitogen-activated protein kinase kinase kinase 1 | MAP3K1_E81 | 0.057 | (0.019) | 0.003 | 0.917 |
| Matrix metallopeptidase 14 | MMP14_P208 | 0.031 | (0.011) | 0.006 | 0.917 |
| Peptidase inhibitor 3, skin-derived | PI3_P1394 | 0.034 | (0.013) | 0.008 | 0.917 |
| Surfactant protein C | SFTPC_E13 | -0.028 | (0.011) | 0.010 | 0.917 |
| Secreted phosphoprotein 1 | SPP1_E140 | -0.042 | (0.017) | 0.014 | 0.917 |
| Runt-related transcription factor 3 | RUNX3_P393 | 0.028 | (0.012) | 0.018 | 0.917 |
| EPH receptor A2 | EPHA2_P203 | -0.038 | (0.017) | 0.026 | 0.917 |
| Excision repair cross-complementing rodent repair deficiency, complementation group 3 | ERCC3_P1210 | -0.034 | (0.011) | 0.003 | 0.917 |
|  |  |  |  |  |  |

Mean difference estimated using linear mixed models. All models adjusted for child’s sex and maternal age at child’s birth and the inclusion of a random batch effect.

Suffixes denote the Illumina probe identity (P = within promoter, E = within first exon).

| **Gene name** | **CpG site** | **Mean (se) difference in Illumina beta value** | | **p value** | **q value** |
| --- | --- | --- | --- | --- | --- |
|  |  |  |  |  |  |
| Egf-like module containing, mucin-like, hormone receptor-like 3 | EMR3_P39 | -0.029 | (0.010) | 0.004 | 0.999 |
| Growth factor receptor-bound protein 7 | GRB7_P160 | -0.035 | (0.014) | 0.018 | 0.999 |
| Hepsin | HPN_P823 | -0.032 | (0.014) | 0.023 | 0.999 |
|  |  |  |  |  |  |

**Table S3.** List of CpG sites with differential methylation at a p value<0.01 per 400g of weight gain/week in late pregnancy (from 29 weeks onwards), the ALSPAC cohort (n=88).

Mean difference estimated using linear mixed models. All models adjusted for child’s sex and maternal age at child’s birth and the inclusion of a random batch effect.

Suffixes denote the Illumina probe identity (P = within promoter, E = within first exon)
